# The associated network embedded decision-making authority allocation and risk-taking of enterprise groups

**DOI:** 10.1371/journal.pone.0318983

**Published:** 2025-05-08

**Authors:** Ning Wang, Fengjuan Wang

**Affiliations:** 1 Business School, Beijing Technology and Business University, Beijing, China; 2 School of Digital Finance, Beijing Institute of Economics and Management, Beijing, China; Southwestern University of Finance and Economics, CHINA

## Abstract

This research examines the impact of the internal allocation of decision-making authority on the risk-taking behavior of enterprise groups, based on a sample of A-share listed parent-subsidiary companies from 2010 to 2022. The study finds that a centralized decision-making structure is associated with reduction in the overall risk profile of enterprise groups. This paper further employs a mechanism analysis through the establishment of a moderated mediation model with an associated network, which reveals that the centralization of internal decision-making authority achieves a reduction in the level of risk-taking through the operation of the internal capital market through two distinct mechanisms. Firstly, it operates the internal capital market, which suppresses diversified investments. Secondly, it improves the efficiency of internal capital allocation. Furthermore, when the associated network is embedded in the internal capital market model, the moderating effect demonstrates that the internal interlocking director network does not suppress diversified investments, but significantly enhances the efficiency of the internal capital market. The associated network of institutional investors serves to reinforce the risk-reducing effect of the internal capital market on risk by reducing noise trading and signal transmission through the diversification and efficiency enhancement of the internal capital market. This paper illuminates the characteristics of network relationships within the internal capital market and the impact of decision-making authority allocation on enterprise risk, and provides strategies to prevent and mitigate significant economic and financial risk.

## 1. Introduction

Enterprise groups constitute over 99% of A-share listed companies in China [1]. In a centralized decision-making structure, authority is concentrated at the upper management of the enterprise group. This approach mitigates the accumulation of risks across organizational units, facilitating the establishment of inter-departmental networks in key functional areas such as business, finance, and technology [2]. This network serves to enhance their ability to withstand external risks and address internal challenges. The interconnections between group members exert a considerable influence on the proclivity to assume risks. Empirical research and case studies [3–5], have identified a positive correlation between the richness of structural holes within the interlocking network and the propensity to take risks. The strategic distribution of decision-making authority within the network fosters technical exchange and cooperation among subsidiaries and with external partners, thereby enhancing the ability of enterprise groups to withstand external risks and address internal challenges. The allocation of resources among departments is contingent upon both productivity and risk-taking decisions, with companies potentially divesting production units to enhance liquidity [6]. This approach not only enhances the efficiency of decision-making processes but also optimizes resource allocation by exploiting the synergistic advantages of pooled resources. However, decision-making authority allocation ensures the consistency of policy and mitigates the proliferation of losses that can result from self-interested actions. The intricate nature of knowledge and the diversifying demands on capital present significant operational challenges for corporate innovation. The integration of the associated network significantly substantially influences the allocation of decision-making authority within enterprise groups, thereby affecting their ability to capitalize on external opportunities and engage in risk-taking. Consequently, examining risk-taking through the lens of network integration has profound implications for unravelling the dynamics of corporate strategy and innovation in the financial sector.

A nation’s economic prosperity and development are contingent upon a robust and thriving real economy. It is imperative that enterprises leverage the internal capital market in an effective manner to guarantee the continuity of their operations and to stimulate innovative development. Inefficiencies in internal capital markets can be addressed through the implementation of relational and contractual control mechanisms [7]. It has been demonstrated that market risk increases in line with the level of income diversification. This can be observed in the case of large microfinance institutions, which have been shown to effectively manage their credit risk [8].

On the one hand, the consolidation of internal power through business alliances and financial network fosters the stability and efficiency of the internal capital market [9–11]. This consolidation optimizes the allocation of resources, eases financial constraints, and reduces the enterprise’s overall risk-taking propensity [12]. Conversely, in the capital markets, enterprise groups often exploit a multitude of capital transactions and related-party transactions to transfer high-value resources from listed firms to their non-listed subsidiaries [13], thereby increasing the groups’ overall risk-taking level [14,15].

On the other hand, the interconnectedness of the network facilitates the integration of internal capital with external funding sources [16]. This integration enables listed companies to secure debt financing [17], which in turn influences the scale, cost, and term structure of the financing [18], and consequently, it enhances the enterprises’ risk-taking capacity. As enterprise groups contend with the dual challenges of internal and external risks, the strategic allocation of decision-making authority has the potential to exert a significant influence on the impact of the internal capital market on risk [19]. This influence extends to the real economy and the entire financial system, underscoring the theoretical and practical significance of this research endeavor.

The extant theoretical and empirical studies are primarily concerned with enhancing the efficiency of investment and reducing the risk for individual enterprises through the optimization of the allocation of decision-making authority at the corporate level. However, there is a paucity of research that considers enterprise groups as a whole. In particular, scholars have been concerned with the impact of equity concentration [20,21] insider shareholding [22], and the legal and regulatory environment on decision-making risk preferences [23]. Secondly, existing literature has also explored the impact of corporate decision-making authority allocation on the efficiency of the internal capital market. There is a consensus that a sound governance structure can help reduce ineffective capital investment between departments. Furthermore, the diversity of a company’s governance structure has been identified as a significant factor affecting its internal capital market and investment efficiency [24–26]. However, previous studies have analyzed the efficiency of internal capital investment from the perspective of centralization and decentralization. They have often overlooked the mutual influence of internal network relationships and have not fully explored the extent of their impact from both internal and external governance perspectives. In conclusion, the majority of existing literature concentrates on enhancing the efficiency of the internal capital market, viewing this as the primary objective of group management [27,28]. However, it frequently overlooks the potential shifts in risk attitudes that may accompany such improvements, which could result in the underestimation of the impact of risk contagion and risk dispersion.

In response to the existing research gaps, our study makes a significant contribution to the literature by integrating the internal decision-making structure, risk-taking behaviors, and characteristics of network relationships within enterprise groups. This provides a new theoretical framework and empirical evidence of understanding how enterprises make decisions in complex environments. The paper presents a meticulous examination of a cohort of A-share companies listed in Shenzhen’s stock market from 2010 to 2022. It adopts a novel approach, analyzing the enterprise group’s associated network and employing social network analysis techniques to construct a model elucidating the impact of internal capital market decision-making on risk-taking behaviors. Furthermore, this research delves into the regulatory effects of both internal and external networks associated with enterprise groups.

This paper’s scholarly contributions are manifold: Initially, it provides an in-depth analysis of the internal capital market’s functioning and risk management mechanisms, focusing on how decision-making authority is allocated within enterprise groups. This is a dimension that has been overlooked in the current literature despite extensive discourse on equity concentration and risk-taking. Subsequently, the research methodology is innovative. By employing social network analysis, this study investigates the interplay between the internal capital market and risk. The incorporation of network-related indicators and the consideration of transactions within unlisted firms introduce a pioneering perspective for examining the propagation and distribution of risks across enterprise groups.

## 2. Methods

### 2.1 Theoretical analysis and research hypotheses

#### 2.1.1 Theoretical analysis and proposition of benchmark regression hypotheses.

The distribution of decision-making authority represents a fundamental aspect of corporate governance, influencing the manner in which enterprises allocate resources. The extant literature understands the allocation of decision-making authority from three principal perspectives: (1) The allocation of decision-making authority is a process and an outcome of the game between various entities within the enterprise [29]. (2) The allocation of decision-making authority plays a pivotal role in determining the distribution of key resources within the enterprise. Rajan (1998) [30] posits that power is derived from control over key resources; [3] The allocation of decision-making authority is the relationship between decision management rights and decision control rights. Fama and Jensen (1983) [31] conceptualize the allocation of decision-making authority as the process of combination and separation of the two types of control rights and decision-making authority. Within enterprise groups, this authority can be categorized into two distinct models: a centralized model and a decentralized mode. The centralized model consolidates decision-making authority, including control over personnel, management, and financial matters, within the parent company. In contrast, the decentralized model disperses decision-making authority among subsidiaries, thereby facilitating greater operational autonomy and strategic flexibility.

The term “risk taking in enterprises” is used to describe the inclination towards high-risk, high-reward investment projects, which reflect the entity’s risk appetite. Research indicates that an elevated level of risk-taking can accelerate the accumulation of shareholder wealth. However, it also increases the overall risk level of the enterprise. Therefore, it is necessary to adopt a balanced approach to risk-taking [32]. A balanced approach to risk-taking is essential, as it provides effective incentives for enterprises while helping to maintain stable operations and avoid potential losses due to excessive risk exposure.

It is proposed that centralized decision-making within enterprise groups will result in the alignment of strategic objectives, enhanced decision-making efficiency, reinforced risk control, and a reduction in agency costs. Specifically, centralized decision-making implies a more rapid and coherent process, enabling prompt responses to market changes and curbing decision-making delays and redundancies [33]. Notable improvements have been observed in the intensity of group operations, credit intensity, innovation in internal financing, and financial service models under centralized management, thereby enhancing decision-making efficiency [34]. Additionally, centralized decision-making is believed to bolster the group’s financial management and risk management capabilities, strengthen centralized control, reduce agency costs, and facilitate a rapid response to external challenges [35]. Noted by international scholars, internal capital markets become particularly dynamic during economic recessions. This is evidenced by the rapid growth of intra-group loans, which are pivotal in aiding enterprises to navigate economic hardships [19]. In consideration of these insights, the following hypothesis is proposed:

**Hypothesis 1 Enterprise groups that adopt a centralized allocation of decision-making authority will demonstrate a diminished level of enterprise risk-taking.**

#### 2.1.2 The analysis and mechanism regression hypothesis proposition.

***2.1.2.1 Decision-making centralization, internal capital market and risk-taking***. Peyer and Shivdasani [36] define the internal capital market as a mechanism for the allocation of funds among the various departments within an enterprise. The internal capital market plays a pivotal role in alleviating external financing constraints and improving resource allocation. The prevalence of information asymmetry and the increase in transaction frictions impose significant external financing costs on firms, while the internal capital market can swiftly meet capital requirements, thereby supporting the company’s expansion and enhancing its financial flexibility [37]. Empirical research also supports the conclusion that, for instance, intragroup reinsurance (a substitute for capital) activities in the U.S. non-life insurance sector can reduce the insolvency risk of member companies, thereby facilitating their pursuit of business growth during market turbulence [38]. In China’s group internal capital markets, there is also the phenomenon of subsidizing zombie enterprises to prevent risk contagion and avoid losing control [15].

From the perspective of the internal capital market’s mechanism of action, a greater centralization of internal power is more conducive to enhancing the efficiency of the internal capital market. The theory of managerial power posits that managers are driven and capable of leveraging their authority to pursue rent-seeking behaviors. In the context of intense competition, the formation of internal capital for competition prompts managers to strategically leverage their domain knowledge and expertise to underreport project cost estimates, thereby achieving optimal resource allocation [39]. Conversely, the higher the degree of centralization within a firm, the stronger the rent-seeking ability of management, which can impede the activity of the internal capital market and lead to a preference for more “prudent” and “favorable” projects [40]. According to agency theory, enterprise managers, considering interests such as compensation, tend to avoid risk and are reluctant to invest in high-risk projects with a positive NPV, thereby reducing the overall risk level. This is particularly the case when decision-making authority is concentrated in a few individuals or a single entity, which can result in a narrow perspective that fails to recognize the potential benefits and necessity for diversified investments [41].

Simon (1947) [42] first proposed the theory of centralization and decentralization, which suggests that enterprises form decision-making units at different levels within the organization. The design of an organization should consider the combination of centralization and decentralization, and establish clear boundaries between the two. Williamson (1975)‘s [43] proposed a transaction cost theory that is consistent with the theory of centralization and decentralization. This theory suggests that organizations have two primary decision-making mechanisms: market and internal authority. U-form organizations adopt an authoritative approach, H-form organizations adopt internal market competition mechanisms, and centralized-decentralized organizational structures (M-form organizations) combine headquarters authority with market mechanisms in their control systems. The integration of Simon and Williamson’s theories can be further applied to the allocation within the internal capital market. This integration allows for the optimization of decision-making mechanisms, the reduction of rent-seeking issues within the internal capital market under a decentralized model, and the reduction of corporate risk-taking.

**Fig 1:**
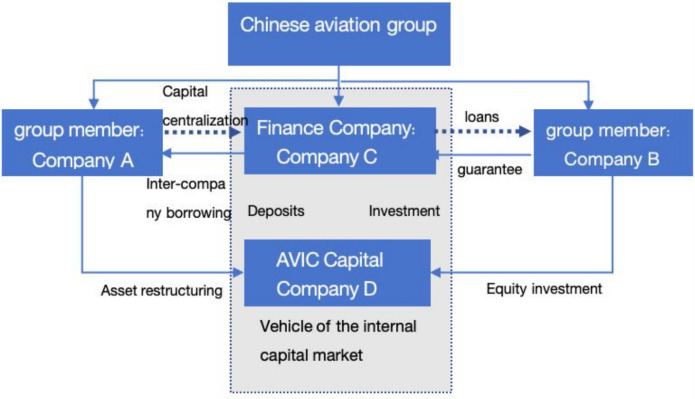
The internal capital market decision-making and operation of China National Aviation Group.

In China, the majority of enterprise groups adhere to a pyramidal organizational structure, which exhibits the defining characteristics of M-form organizations. The allocation of resources is conducted through the function of the internal capital market, which serves as a decision-making apparatus integrating the authority of the headquarters with market mechanisms. Fig 1 depicts the decision-making and resource allocation processes of a Chinese state-owned aviation group. The Chinese aviation group is an example of an M-form organizational structure, with more than 400 subsidiaries. The internal capital operation platforms are the China Aviation Finance Company and AVIC Capital (see company A and B). These group member companies engage in daily centralized business with Finance Company C and conduct internal borrowing, entrusted loans, and guarantee business, thus forming an inter-company capital association network. Conversely, AVIC Capital D enables the group to expand externally while simultaneously restructuring internally, thereby facilitating the formation of a diversified internal business capital operation. Companies C and D engage in deposit and investment activities pertaining to fund transactions. The AVIC Group, through the centralization of capital operations and decision-making at the headquarters level and the consolidation of compensation and equity in Finance Company C and Capital Company D, is able to mitigate overall enterprise risk. The concentration of corporate power facilitates the allocation of significant resources to the internal market, thereby reducing the overall risk undertaken by the group.

Therefore, based on internal capital market theory, the following hypothesis is proposed:

**Hypothesis 2.1 The centralized allocation of decision-making authority has the effect of reducing the overall level of risk-taking, due to the limitation of intermediary functions in the diversified operations of the internal capital market.**

**Hypothesis 2.2 The centralized allocation of decision-making authority within enterprise groups has the effect of reducing the overall level of risk-taking, due to the enhancement of the intermediary role in improving the efficiency of internal capital market allocation.**

***2.1.2.2 Associated interlocking director network and the internal capital market under enterprise centralization***. The interlocking director network is a network structure formed by company directors based on common stock ownership, alumni relationships, social connections, and various other means. The existence of this network allows directors to influence each other, share information, and play a role in corporate governance. Enterprise groups often establish an interlocking network of board members that extends to outside companies. Under centralized management, interlocking directors can have a significant impact on internal capital decisions through their network, contributing to monitoring, information dissemination, and governance mechanisms. The governance effects of interlocking directors can manifest themselves as:

**The suppressive effect of interlocking director network on the diversification of internal capital market operations.** For the internal capital market to thrive, it requires not only the rational allocation of internal resources but also effective interaction with the external market. Existing literature confirms that the operation of internal capital markets is greatly influenced by corporate governance. In the study by Autner and Villalonga [44], they used the exogenous shock of the German tax reform on corporate ownership structure and found that companies with more concentrated ownership are less diversified, while having more efficient internal capital markets. The misallocation of internal capital is partly due to poor corporate governance. Moreover, from the perspective of mitigating information asymmetry, the information content of a company’s stock price is positively correlated with its capital investment efficiency, and a good director decision-making mechanism plays a positive moderating role [45].

**The enhancing effect of interlocking director network on improving the efficiency of internal capital markets.** Under a centralized framework, these related directors can exert significant influence over internal capital decisions through their network, contributing to monitoring, information dissemination, and governance mechanisms. When the director network exhibits a high degree of centrality, network members are more likely to conform, creating a collective effect and establishing a network governance dynamic [46]. At the same time, the network of related directors can mitigate issues of information asymmetry by providing enterprises with precise market and industry insights. This network can also help alleviate financing constraints, improve internal investment efficiency, and streamline diversified operations [47].

**Hypothesis 3.1: The network of interlocking directors can intensify the suppressive effect of centralized decision-making authority on the diversification of internal capital market operations.**

**Hypothesis 3.2: The network of interlocking directors further enhances the effect of centralized decision-making authority within enterprise groups on improving the efficiency of the internal capital market.**

***2.1.2.3 Internal capital market and risk-taking within associated network***. The network of affiliated institutional investors is a network structure formed by institutional investors based on common stock ownership or other related relationships. Compared to interlocking directors, it is more difficult for affiliated institutional investors to influence key decisions in the internal market of the company. The ways in which institutional investors typically participate in corporate governance are: first, by exiting, or voting with their feet, selling their shares; and second, by voicing their opinions, continuing to hold their shares [48]. The role in the internal capital market in terms of risk disclosure is manifested as follows:

The network of affiliated institutional investors can further strengthen the role of reducing internal capital diversification in reducing risk taking. On the one hand, the network connections and information sharing among affiliated institutional investors can reduce the diversification within a company, as investors tend to concentrate resources on investments that are widely recognized by the market and have lower risks [49]. On the other hand, by reducing noise trading, these investors can reduce the company’s risk exposure [50]. The signal transmission function of institutional investors’ increases and decreases in holdings is significant; when institutional investors acquire hidden bad news about management, their actions can trigger a “herding effect” among affiliated institutional investors, further reducing the effect of internal capital market diversification on risk reduction. This mechanism can prompt company management to reduce unnecessary diversification investments and avoid the uncontrolled risk expansion.

The network of affiliated institutional investors can further enhance the role of improving internal capital efficiency in reducing risk-taking. Through their reputation mechanisms and signal transmission functions, the network of affiliated institutional investors can improve the efficiency of the internal capital market and educe the risk-taking effect of enterprises. Empirical studies of listed companies in China indicate that the efficiency of the institutional investor network has a significant impact on market information efficiency [51]. Signals transmitted through the network by institutional investors can influence corporate behavior and market performance, thereby improving the efficiency of the internal capital market and reducing risk [52].

**Hypothesis 4.1 The network of affiliated institutional investors can further strengthen the role of reduced internal diversification in reducing risk-taking.**

**Hypothesis 4.2The network of affiliated institutional investors further enhances the effect of improving internal capital market efficiency on risk reduction through a reputation mechanism.**

**Fig 2:**
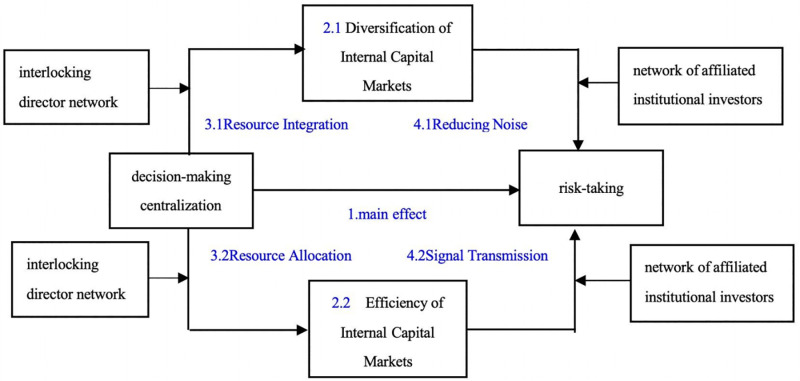
Corporate group decision-making and risk effects based on internal affiliation network.

In summary, Fig 2 illustrates the pathways through which corporate centralization affects risk-taking, with a total of four sets of hypotheses, namely that the internal capital market acts as a mediator, and the effect is intensified through the associated network of corporate governance.

### 2.2. Research design

#### 2.2.1. Sample selection and data source.

To mitigate the impact of the 2018 financial crisis on stock price dynamics, this study focuses on the annual observations of 16,997 listed companies from the Shanghai and Shenzhen stock exchanges and their subsidiaries over the period from 2010 to 2022. The sample excludes cases with incomplete data, financial industry firms, and outliers. In addition, continuous variables were truncated at the 1% upper and lower quantiles to reduce the impact of extreme values. The financial data of the sampled companies were obtained from the CSMAR database. For the relevant data, please refer to Surporting information S3 Data. The internal capital market of enterprises was constructed based on inter-group related party transactions, with transaction data sourced from the CCER database. Additionally, an associated director network was built based on the interlocking directorships across different listed companies, and an associated investor network was established based on the holdings of the same institutional investor in different listed companies. Gephi software was used to calculate network metrics and centrality measures, allowing for a nuanced examination of the network structures and their potential influence on corporate decision-making and risk profiles.

#### 2.2.2. Variable definitions.

***2.2.2.1 Interpreted variables: risk-taking***. A proclivity for high-risk decision-making can precipitate operational crises and financial risks for companies, which in turn can impede a company’s growth and development. The academic community currently employs three principal methods for measuring the extent of corporate risk-taking. Firstly, t fluctuations in performance must be considered. The returns associated with high-risk investment projects also exhibit considerable volatility. Secondly, the level of innovation and research and development intensity must be considered. Corporate investment in innovation and R&D has been demonstrated to exert a positive influence on the level of risk-taking. Thirdly, the probability of corporate survival. In general, the shorter the duration of a company’s existence, the higher the probability of its bankruptcy. This paper primarily examines the impact of corporate decision-making on risk-taking. Fluctuations in market value can better reflect the scope of decision-making influence and can more accurately depict the external impacts on the internal capital market. The dependent variable in this study is the overall risk, encompassing both systemic and idiosyncratic risks. To ensure t clarity and readability of the empirical results, the discussion of systematic risk and idiosyncratic risk is placed within the analysis of heterogeneity. To estimate the overall risk level, denoted as R_i,t_, we follow the methodology proposed by Bernile, G. [53,54]. Specifically, we consider the daily return rate of stock i, including the reinvestment of cash dividends on day t, and adjust this rate to reflect an annualized figure by multiplying by 250 and applying a logarithmic transformation, resulting in the variable ‘risk’.

***2.2.2.2 Interpretive variables: decision-making configuration (CI)***. The interpretive variable for decision-making configuration, denoted as CI, is used to measure the degree of the centralization of decision-making authority within an enterprise group. This index is derived from the concentration of compensation, which serves as an indicator of the degree of control exerted by the parent company over subsidiary decisions. The centralization index is based on the relative rigidity of contracts, which is exogenous to the subsidiary’s managerial compensation, and is directly related to the subsidiary’s development. This approach allows for a more accurate reflection of the characteristics of the decision-making configuration [55]. The calculation of CI is primarily informed by the work of Wang Liang-liang, who calculated the centralization degree using the following equation:


PSalaryit=β0+β1PAssetit+εit.

In this equation, *PSalary*_it_ represents the proportion of employee compensation paid by the parent company. This is measured as the cash paid in the parent company’s cash flow statement divided by the corresponding items in the consolidated financial statements. The proportion of the parent company’s assets, denoted by *PAsset*_it_, is calculated as the total assets of the parent company divided by the total assets in the consolidated financial statements. To mitigate the impact of outliers, both *PSalary*_*it*_ and *PAsset*_*it*_ are capped within the interval [0,1], and samples with negative net assets in the consolidated statement are excluded. Annual regressions are conducted by industry, and the estimated residual from this regression (CI) is used as a measure of the degree of centralization of decision-making authority. A higher value of the CI index indicates a higher degree of centralization within the enterprise group.

***2.2.2.3 Intermediary variables: internal capital market***. In the extant literature, the construction of the internal capital market is typically gauged by indicators such as the ratio of related-party funds to total assets and the magnitude of internal cash flows. This study proposes that the extent of corporate diversification serves as a reliable indicator of the scale of the internal capital market. In this study, we employ the concept of corporate diversification, as proposed by Lu Jian-xin and other scholars [56], to represent the internal capital market’s development. The revenue entropy, denoted as Dhy,is utilized as the primary measurement index. The formula for calculating revenue entropy is given by:Dhy =∑*P*_i_* × ln(1/P*_i_), In this formula, Pi represents the proportion of the main business income of category ii relative to the total business income. A higher value of revenue entropy indicates a more active internal capital market, suggesting a greater diversity of investment opportunities and a more efficient allocation of resources within the enterprise.

The vitality of the internal capital market is reflected in the allocation of resources, measured by the extent of internal funding transactions within the enterprise. This is defined as the sum of the enterprise’s internal related-party payables and receivables, standardized by total assets. The efficiency of the internal capital market is a better indicator of whether resource allocation is reasonable and effective. This paper refers to Richardson’s [57] approach to constructing investment efficiency, utilizing a model to predict internal capital market transactions within enterprises and subsequently reflecting the efficiency of the internal capital market in terms of the degree of deviation. The prediction model is as follows:


$$Icm_{,t}=\;\;\alpha_{0}+\alpha_{1}Icm_{i,t-1}+\alpha_{2}TobinQ_{i,t-1}+\alpha_{3}Cash_{i,t-1}+\alpha_{4}Lev_{i,t-1}+\alpha_{5}Size_{i,t-1}+\alpha_{6}Roa_{i,t-1}+\alpha_{7}IPO_{i,t-1}+\lambda_{i}+\;+\;\mu_{t}+\varepsilon_{i,t}$$

In this model, the variable Icm_i,t_ represents the current internal related transaction amount, the variable TobinQ_i,t-1_ is the enterprise’s Tobin’s Q value at the beginning of the year (calculated as the proportion of market value to total assets at the beginning of the year), and the variable IPO_i,t-1_ represents the number of years the enterprise has been listed. λ_i_ and μ_t_ are the individual and time fixed effects, respectively. ε_i,t_ represents the residual of the model, indicating the degree of deviation of the internal capital market from predicted outcome. In order to enhance data readability and align with the established definition of investment efficiency, the residual is standardized to a 0–1 scale. Consequently, an elevated Icme signifies a higher level of efficiency within the internal capital market.

***2.2.2.4 Adjustment variables: associated network***. In the context of network centrality measures, previous studies have predominantly employed three indicators: degree centrality, closeness centrality, and betweenness centrality [58]. This paper has selected closeness centrality because this indicator emphasizes the “proximity” or “reachability” of a node to other nodes, which more effectively reflects the efficiency of information transfer within the internal capital market. In contrast, degree centrality emphasizes the number of direct connections a node has, whereas betweenness centrality highlights the node’s intermediary role within the network. The formula for calculating closeness centrality (CC(i)) is as follows:


$$CC\left(i\right)=\frac{1}{\sum d\left(i,j\right)}$$

In the formula, d(i,j) represents the length of the shortest path from node i to node j,and j≠i

**Fig 3:**
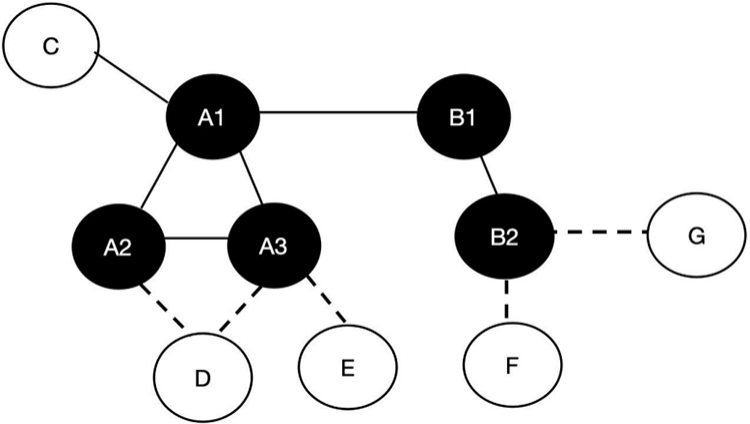
Calculation of Key Indicators in the Associated Network.

As illustrated in Fig 3, the following companies are members of the group: A, B, C, D, E, F, and G. A1, A2, and A3 are directors or institutional investors of Company A, while B1 and B2 are directors or strategic investors of Company B. In the event that A1 also serves as a director of Company B, or B1 serves as a director of Company A, the closeness centrality of A1, denoted as CC(A1), can be calculated as follows: CC(A1) = 1/10*(4 + 2*3 + 3*3)=1/1.9%, which is greater than the closeness centrality of B1,denoted as CC(B1) = 1/10*(2 + 2*6 + 2*3)=1/2%.Therefore, A1 has more channels to access resources and information.

The term “interlocking director network” is used to describe the inter-enterprise relationship network established by directors who serve on the boards of two or more companies due to their cross-directorships, whether directly or indirectly. The network relationship indicators are represented by the network of associated directors, which is denoted as ‘Network_d’. The calculation of this index is facilitated by Gephi software, which is based on the manual organization of director information. The process comprises several steps. Initially, director data is meticulously compiled and cleansed. Subsequently, a matrix is constructed to establish adjacencies based on recurring directors. Ultimately, the proximity between a director and other directors within the network is determined using the defined and identified chain director data. A higher centrality score indicates a director’s closer connection to other directors, signifying a more significant advantage in information acquisition and dissemination.

The term “institutional investor network” is used to describe the connections formed between institutions through the sharing of stockholding relationships. Such a network can effectively reflect the various social relationships that exist among individual institutional investors. Constructed using the same method, if two institutional investors hold at least a combined percentage of a company’s outstanding shares that is equal to or greater than 5% by the end of the year, then a connection between these two institutions is considered to be established. A matrix is constructed in a manner similar to that employed for interlocking directors, indices are calculated, and denoted as”Network_i”. The higher the closeness centrality (Network_i and Network_d), more readily accessible are resources within the network, and the connections with numerous other nodes are more robust.

***2.2.2.5 Control variables.*** This study incorporates controls for both annual fixed effects and individual fixed effects at the company level to account for unobserved heterogeneity that could influence the relationship between decision-making authority allocation and risk-taking.

Table 1 presents the definitions and descriptions of the primary variables utilized in the analysis, ensuring a comprehensive understanding of the data structure and the constructs being examined.

**Table 1:** Definitions and Descriptions of Main Variables.

*variable definition*	*variable symbol*		*variable declaration*
*Interpreted variable*	*risk*		*Total risk: the standard deviation of the annual daily return rate of the listed company*
*Interpretive variable*	*CI*		*Degree of capital centralization within the enterprise group: PSalaryi,t=β0+β1PAsseti,t+ε The model shown performs annual regression and takes the estimated residual as a measure of the degree of centralization*
*mediating variable*	*Icm*	*Icm*_*d*_	*Operation mode of internal capital market: revenue entropy index* *Icm*_*d*_* = P*_*i*_*ln (1/ P*_*i*_*), P*_*i*_* = main business income/total business income of enterprise category I. The larger the Icm*_*d*_*, the more diversified the internal capital market is.*
		*Icm*_*e*_	*Efficiency of internal capital market within the enterprise group Icm*_*ei,t*_*=α*_*0*_*+α1Icm*_*ei,t − 1*_*+α*_*2*_*TobinQ*_*i,t − 1*_*+α*_*3*_*Cash*_*i,t − 1*_*+α*_*4*_*Lev*_*i,t − 1*_*+α*_*5*_*Size*_*i,t − 1*_*+α*_*6*_*Roa*_*i,t − 1*_*+α*_*7*_*IPO*_*i,t − 1*_*+λ*_*i*_* + μ*_*t*_*+∊*_*i,t*_ *and the larger the absolute value, the higher the efficiency.*
*Adjustment* *variables*	*Network*	*Network*_*d*_	*The proximity of the director network reflects the proximity between a director and other directors in the network. For a director, the closer he is to other directors, the greater the proximity center, and the more obvious the information advantage between the directors*
		*Network*_*i*_	*The proximity of the institutional investor network reflects the proximity between an institutional investor and other institutional investors in the network. For an institutional investor, the closer it is to other institutional investors, the greater the proximity center, and the more pronounced the information advantage between the institutional investors.*
*Control* *variables*	*Size*		*Total assets taken to the natural logarithm.*
	*TobinQ*		*Total market value/ assets*
	*Growth*		*Current operating income/ previous operating income of-1*
	*lev*		*Interest-bearing liabilities/ total assets*
	*roa*		*EBITDA/ total assets*
	*top1*		*The largest shareholder shareholding ratio*
	*BoardScale*		*The board of directors’ size (number of members)*
	*EU*		*The standard deviation of the residuals of operating income adjusted for the industry over the past 5 years*
	*Year*		Annual virtual variable
	*Industry*		Industry virtual variable

### 2.3. Model construction

This study constructs empirical models to investigate the influence of enterprise group control on enterprise group risk. The models are specified as follows:


$$Risk_{i,t}=\alpha_{0}+\alpha 1CI1\;CIit+\alpha_{2}X_{it}+\sum Industry+\varepsilon_{i,t}Model$$ (1)

$$Icm_{i,t}=\beta_{0}+\beta 1CI1\;CIit+\beta_{2}Network_{it}+\beta_{3}Icm_{it}\times Network_{it}\text{+}\beta_{4}X_{i,t}\text{+}\sum Year+\sum Industry+\varepsilon_{i,t}Model$$ (2)

$$Risk_{i,t}=\beta_{0}+\beta_{1}Icm_{it}+\beta_{2}Network_{it}+\beta_{3}Icm_{it}\times Network_{it}\text{+}\beta_{4}X_{i,t}\text{+}\sum Year+\sum Industry+\varepsilon_{i,t}Model$$ (3)

A benchmark model (1) is established to test the impact of centralized management (CI) on risk-taking (Risk). Model (2) is the regulatory mediation model, which examines the internal capital market (Icm) operation model under the influence of enterprise group centralized management (CI) and network relationships (Network). The model incorporates the moderating effects of the associated director network (Network_d) and the associated investor network (Network_i) on the operation of the internal capital market (Icm). The functioning of the internal capital market is manifested in two key aspects: the diversification of the internal capital market (Icm_d) and the efficiency of resource allocation within the internal capital market (Icm_i). Model (3) is a regulatory mediation model that tests the risk effect of the internal capital market (Icm_d and Icm_e) with the network relationship acting as the regulatory factor influencing risk conduction (Risk). In these models, the subscripts i and t represent the enterprise and time period, respectively. X_i,t_ represents a vector of control variables, including those previously described earlier for enterprise characteristics. ∑ Year denotes the fixed effect for each year, while *∑Industry* represents the fixed effect for each individual enterprise. ∊_i,t_ signifies the random disturbance term.

## 3. Results and discussion

### 3.1. Benchmark empirical results and analysis

#### 3.1.1 Descriptive statistical analysis.

Table 2 provides the basic statistical overview of the variables utilized in this study. The centralization index (CI), which serves as the primary explanatory variable, exhibits a mean value of -0.01. This finding is consistent with existing literature, which suggests a tendency for enterprises to adopt a decentralized power structure. The distribution of CI values is notably left-skewed, with a minimum of -0.216 and a maximum of 0.171, indicating a considerable range in the degree of centralization across the sample.

**Table 2:** Descriptive Statistics Table.

Variable	Obs	Mean	Std. Dev.	Min	Max
risk	16997	1.892	0.29	1.146	2.589
CI	16997	-0.01	0.082	-0.216	0.171
Icm d	16997	0.6	0.474	0	1.466
Icm e	16997	0.683	0.156	0.458	0.997
Network d	16997	0.133	0.043	0	0.186
Network i	16997	0.211	0.359	0	1
size	16997	22.612	1.329	20.084	26.413
roa	16997	0.039	0.055	-0.183	0.198
top1	16997	0.355	0.15	0.096	0.75
lev	16997	0.461	0.196	0.074	0.885
growth	16997	0.331	0.921	-0.662	6.521
BoardScale	16997	8.717	1.693	5	15
tobin	16997	1.96	1.265	0.834	8.224
EU	16997	1.252	1.383	0.083	8.446

The core dependent variable, “risk,” with a mean of 1.892 and a standard deviation of 0.29, indicates that the average level of risk across the sample companies is relatively high, with some variation in risk exposure. The range from a minimum of 1.146 to a maximum of 2.589 indicates a considerable divergence in risk levels, suggesting that while some companies exhibit a relatively risk-averse disposition, others demonstrate a proclivity for assuming greater risks. The mean value of the internal capital market diversification (Icm d) has a mean of 0.6, indicating a moderate level of diversification in internal capital market operations. The mean value of the internal capital market efficiency (Icm e) is 0.683, suggesting that the efficiency of resource allocation within the internal capital market is relatively high. However, there is considerable variation, as evidenced by the standard deviation of 0.156.

The network characteristic variables align with the current body of literature, exhibiting means of 0.133 and 0.211, respectively. This indicates that directors occupy distinct positions within the network, which affects their capacity to access resources, information, and their governance roles. The selection of other control variables reflects mainstream academic consensus, ensuring the robustness of the study’s methodology.

#### 3.1.2 Analysis of benchmark regression results.

Table 3 presents the results of the benchmark regression analysis, which examines the relationship between the centralization of decision-making authority and the risk levels within enterprise groups. As evidenced by the results in columns (1) and (2), the coefficients for the centralization index (CI) are -0.065 and -0.175, respectively, both of which are statistically significant at the 1% level. This indicates that the concentration of decision-making authority can significantly reduce the risk-taking of enterprise groups, thereby Hypothesis 1. This suggests that as the degree of centralization in decision-making authority increases, the overall risk level of enterprise groups significantly decreases. This highlights the importance of considering the allocation of decision-making authority when assessing the risk dynamics within enterprise groups. In general, a more centralized decision-making structure is associated with a lower propensity for risk-taking in enterprise groups. This may be related to the fact that centralized management can enhance decision-making efficiency and reduce risk exposure.

**Table 3:** Test Results of the Influence of Enterprise Group Decision-making authority on Risk-Taking.

	(1)	(2)	(3)	(4)		(5)	(6)
VARIABLES	risk	risk	risk	risk	VARIABLES	risk	risk
CI	-0.065^***^	-0.175^***^	-0.067^***^	-0.179^***^	L.CI	-0.024	-0.147^***^
	(0.021)	(0.020)	(0.021)	(0.020)		(0.024)	(0.022)
size		-0.052^***^		-0.052^***^	size		-0.050^***^
		(0.002)		(0.002)			(0.002)
roa		-0.095^***^		-0.095^***^	roa		-0.131^***^
		(0.037)		(0.034)			(0.041)
top1		-0.045^***^		-0.045^***^	top1		-0.056^***^
		(0.012)		(0.012)			(0.013)
lev		0.221^***^		0.221^***^	lev		0.245^***^
		(0.011)		(0.011)			(0.013)
growth		0.005^***^		0.005^***^	growth		0.006^***^
		(0.002)		(0.002)			(0.002)
BoardScale		-0.004^***^		-0.004^***^	BoardScale		-0.004^***^
		(0.001)		(0.001)			(0.001)
tobin		0.035^***^		0.035^***^	tobin		0.040^***^
		(0.002)		(0.002)			(0.002)
EU		0.004^***^		0.004^***^	EU		0.006^***^
		(0.001)		(0.001)			(0.002)
Constant	1.891^***^	2.942^***^	2.016^***^	2.965^***^	Constant	1.882^***^	2.880^***^
	(0.002)	(0.042)	(0.029)	(0.047)		(0.002)	(0.046)
Observations	16,997	16,997	16,997	16,997	Observations	14,482	14,482
R-squared	0.446	0.517	0.446	0.517	R-squared	0.452	0.524
Year FE	Yes	Yes	Yes	Yes	Year FE	Yes	Yes
Indu FE	Yes	Yes	Yes	Yes	Indu FE	Yes	Yes

Robust standard errors in parentheses

*** p < 0.01,

** p < 0.05,

* p < 0.1

Furthermore, the results of the regression analysis indicate that factors such as the concentration of decision-making authority (CI), company size (Size), roa, ownership concentration (Top1), financial leverage (lev), growth potential (growth), board size (BoardScale), Tobin’s Q, and environmental uncertainty (EU) significantly affect the risk-taking levels of enterprise groups. These findings highlight the significance of prudent resource allocation and robust governance structures in evaluating corporate risk dynamics. This approach enables organizations to maintain competitiveness and sustainable growth while mitigating risk.

#### 3.1.3 Robustness test.

***3.1.3.1 Instrumental variable method***. Although the primary model [1] draws upon existing literature and employs a two-way fixed effects approach to control for commonalities in the determinants of enterprise group risk, there remains a possibility of bias stemming from omitted variables. Furthermore, potential measurement errors in variables such as the centralization index of decision-making authority could lead to an inadvertent skewing of the study’s conclusions. To mitigate the influence of endogeneity on the findings, this study employs the IV-2SLS method as a control mechanism. In accordance with a common practice in the literature, this paper utilizes the median centralization degree (CIP) of other enterprise groups within the same province and year as an instrumental variable. This choice is based on the assumption that the centralization practices of peer groups can serve as a proxy for the centralization degree of the focal enterprise group, without being directly influenced by the same omitted variables that may affect the dependent variable. Upon performing the two-stage least squares (2SLS) test with the selected instrumental variable, the regression analysis in Table 3 columns (3) and (4) confirms that the initial conclusions remain unchanged. This finding suggests that the observed relationship between the centralization of decision-making authority and enterprise group risk is robust, even after accounting for potential endogeneity.

***3.1.3.2 Explanatory variables lagging behind one phase***. When the explanatory variables are lagged by one period, the fundamental conclusions regarding risk remain robust (see from Table 3). In the benchmark regression, the coefficient for the degree of centralization of decision-making authority (CI) is -0.065 (column (1)) and -0.175 (column (2)), while in the regression with one-period lagged variables, the coefficient for CI becomes -0.024 (column (5)) and -0.147 (column (6)). This indicates that when the explanatory variable is lagged by one period, the impact of CI on reducing risk (risk) is somewhat weakened, particularly in Model 2, where the absolute value of the coefficient increases from -0.175 to -0.147. This suggests that the effect of centralized decision-making on risk reduction is somewhat reduced after a one-period lag. In the benchmark regression, the coefficient for CI is statistically significant at the 1% level, irrespective of whether control variables are included. In the regression with one-period lagged variables, the coefficient for CI remains significant at the 1% level only after control variables are included (indicated by ***). This may suggest that the model is robust to some extent, meaning that the negative correlation between centralized decision-making and risk-taking persists even after a one-period lag.

***3.1.3.3 Replacement of interpretive and interpreted variables***. Enterprise groups constitute a broad internal capital market within listed companies. Centralized control is achieved through a unified control network. Consequently, this paper refers to the existing research practices, The concentration authority concentration within the enterprise group is systematically identified by using the following standards: when two or more listed companies have the same final controller in the same year, these listed companies are considered to be subordinate to enterprise groups [59]. In accordance with the aforementioned, the final controllers of the listed company were manually identified during the sample period. This was done in order to ascertain whether the listed company belonged to the same final controller group (group = 1) or not (group = 0). The existence of an internal capital market was determined on this basis. According to the Altman [60], the financial risk of enterprises is gauged through the Z-score symbol. The index is a composite of financial indicators of listed companies, with the financial data ratios calculated based on their respective weights, and standardized for data readability. An enterprise is considered to have a higher financial risk the greater its Z-score. As illustrated in Table 4 presents the regression analysis results after variable substitution. In columns (1) and (2), with the Z-score as the dependent variable, the coefficients for the centralization index (CI) are -0.338 and -0.694, respectively, both of which are significant at the 1% level.

**Table 4:** Regression Results of the Internal Capital Market and Enterprise Financial Crisis Model.

	(1)	(2)	(3)	(4)
VARIABLES	zscore	zscore	risk	risk
CI	-0.338^***^	-0.694^***^		
	(0.106)	(0.110)		
GROUP			-0.044^***^	-0.020^***^
			(0.004)	(0.004)
size		-0.136^***^		-0.047^***^
		(0.009)		(0.002)
roa		-0.585^***^		-0.134^***^
		(0.185)		(0.037)
top1		0.152^**^		-0.046^***^
		(0.060)		(0.011)
lev		0.035		0.210^***^
		(0.090)		(0.011)
growth		-0.034^***^		0.005^***^
		(0.010)		(0.002)
BoardScale		-0.001		-0.003^***^
		(0.006)		(0.001)
tobin		0.144^***^		0.035^***^
		(0.017)		(0.002)
EU		0.005		0.004^***^
		(0.009)		(0.001)
Constant	4.740^***^	7.487^***^	1.912^***^	2.839^***^
	(0.007)	(0.185)	(0.002)	(0.041)
Observations	16,997	16,997	16,997	16,997
R-squared	0.171	0.227	0.448	0.513
Year FE	Yes	Yes	Yes	Yes
Indu FE	Yes	Yes	Yes	Yes

Robust standard errors in parentheses

*** p < 0.01,

** p < 0.05,

* p < 0.1

These findings indicate that a concentration of decision-making authority is significantly associated with a reduction in corporate financial risk. When compared to the overall risk in the main regression, the reduction effect is more pronounced, suggesting that the centralization of decision-making authority is more effective in reducing financial risk. When the dependent variable is replaced with risk, columns (3) and (4) demonstrate that the coefficients for CI remain significantly negative, at -0.044 and -0.020, respectively. This further confirms that focusing on the concentration of actual controller power within the internal capital market, as compared to the absence of centralized decision-making authority, can more effectively reduce a company’s risk-taking. **Hypothesis 1.**

### 3.2. Mechanism and regulation analysis

This paper further examines the mechanism of centralized management mode to reduce risk-taking. According to the theoretical analysis, this paper will conduct a three-step mechanism test of intermediary variables from decision-making authority allocation, internal capital market and risk-taking, and test the role of correlation network regulation on the mechanism. As the risk of the explained variable is consistent with the results of the specific index risk in descriptive statistics and benchmark regression, the following test shows the overall enterprise risk representing the risk level.

#### 3.2.1. Analysis of the intermediary effect of the internal capital market without associated network.

As previously discussed in the theoretical analysis, corporate centralization can influence a company’s risk-taking through the internal capital market. Companies with centralized decision-making authority optimize the allocation of internal resources and ensure precise investments through the internal capital market. This approach effectively suppresses inefficient investments in both internal and external mergers and acquisitions while simultaneously enhancing the efficiency of internal capital market allocation. Consequently, the impact of enterprise groups on risk-taking will be evaluated through the inhibitory diversification effect and efficiency enhancement effect of the internal capital market.

***3.2.1.1 Inhibitory Diversification Effect*.** Column (2) of Table 3 demonstrates that the total effect of centralized decision-making configuration on corporate risk-taking is significant, thereby establishing the foundation for evaluating the mediating effect. Columns (1) and (2) of Table 5 report the mediating test regression results of the diversification effect of the internal capital market on the relationship between centralized decision-making configuration and corporate risk-taking. The regression results of Model 2 and 3 indicate that the coefficient for centralized decision-making configuration (CI) and internal capital market diversification (icm_d) is -0.307, which is significantly negative at the 1% level. This suggests that enterprise groups with more centralized decision-making authority have lower levels of internal capital diversification. As evidenced by the regression results presented in Column (2), the coefficient for CI is -0.162, while the coefficient for icm_d is 0.043. When the internal capital market is not in the model, the coefficient from X to Y is -0.175 (Column (2) of Table 3). After the addition of the mediating variable icm_d, the coefficient from X to Y becomes -0.162. This means that the absolute value of the direct effect from X to Y is actually weakened after adding Intermediary variables, meeting the requirements for mediating testing. The aforementioned results demonstrate that the diversification of the internal capital market plays a mediating role, thereby Hypothesis2.1 is supported.

**Table 5:** The mediating role of the internal capital market in the relationship between decision-making authority allocation and risk-taking.

	(2)	(3)	(4)	(5)
VARIABLES	icm__d_	risk	icm__e_	risk
CI	-0.307^***^	-0.162^***^	0.178^***^	-0.150^***^
	(0.043)	(0.020)	(0.015)	(0.020)
Icm_d		0.043^***^		
		(0.003)		
Icm_e				-0.144^***^
				(0.010)
size	0.065^***^	-0.055^***^	0.001	-0.052^***^
	(0.004)	(0.002)	(0.001)	(0.002)
roa	-0.538^***^	-0.072^*^	0.007	-0.094^**^
	(0.076)	(0.037)	(0.026)	(0.037)
top1	-0.030	-0.044^***^	-0.075^***^	-0.056^***^
	(0.025)	(0.011)	(0.009)	(0.012)
lev	0.206^***^	0.212^***^	-0.004	0.221^***^
	(0.024)	(0.011)	(0.009)	(0.011)
growth	0.016^***^	0.005^**^	-0.002	0.005^**^
	(0.004)	(0.002)	(0.002)	(0.002)
BoardScale	0.005^**^	-0.004^***^	0.003^***^	-0.003^***^
	(0.002)	(0.001)	(0.001)	(0.001)
tobin	0.013^***^	0.035^***^	-0.006^***^	0.035^***^
	(0.004)	(0.002)	(0.001)	(0.002)
EU	0.008^***^	0.004^***^	0.006^***^	0.005^***^
	(0.003)	(0.001)	(0.001)	(0.001)
Constant	-1.026^***^	2.987^***^	0.665^***^	3.038^***^
	(0.082)	(0.042)	(0.030)	(0.042)
Observations	16,997	16,997	16,997	16,997
R-squared	0.153	0.522	0.074	0.523
Year FE	Yes	Yes	Yes	Yes
Indu FE	Yes	Yes	Yes	Yes

Robust standard errors in parentheses

*** p < 0.01,

** p < 0.05,

* p < 0.1

***3.2.1.2 Efficiency Enhancement Effect*.** The results of the mediating test regression, as reported in columns (3) and (4) of Table 5,report the mediating test regression results of the internal capital market efficiency effect on the relationship between the centralized decision-making configuration and corporate risk-taking. The coefficient for centralized decision-making configuration (CI) and internal capital market efficiency (icm_e) is 0.178, significantly positive at the 1% level. This indicates that enterprise groups with more centralized decision-making authority tend to exhibit higher internal capital market efficiency. As evidenced by the regression results presented in Column (4), the coefficient for CI is -0.150, while the coefficient for icm_e is -0.144. In the absence of the internal capital market in the model, the coefficient from X to Y is -0.175 (Column (2) of Table 3). After the addition of the mediating variable icm_e, the coefficient from X to Y becomes -0.150. This indicates that the absolute value of the direct effect from X to Y is in fact weakened in absolute value following the introduction of the mediating variable, thus meeting the criteria for mediating testing. The aforementioned results demonstrate that internal capital market efficiency plays a mediating role, and Hypothesis2.2 is supported.

#### 3.2.2. Analysis of the impact of decision centralization on internal capital market embedded in interlocking director network.

***3.2.2.1 The moderating effect of the interlocking director network on the impact of decision centralization and internal capital market diversification.*** Table 6, column (1), presents the impact of concentrated de (CI) on the diversification of the internal capital market (Icm_d) after controlling for the effects of the director network. In Model (2), the coefficient for the interaction term c.CI × c.Network_d is 0.155 after centering the variables, and is not significant, indicating that Hypothesis 3.1 is not supported by the data. This may indicate that the effects of concentrated ownership and interlocking director network on the diversification of the internal capital market are not synergistic, or that the impact of interlocking director network varies across different enterprises. Conversely, the coefficient for Network_d is 0.456 and is significant, suggesting that interlocking director network tend to promote diversification. This finding may be related to the ability of interlocking director network to provide broader market and industry information and alleviate issues of information asymmetry, thereby making enterprises more inclined to engage in diversified investments to spread risk and capture more market opportunities. The possible reasons for this are twofold. Firstly, the positive impact of interlocking director network on diversification can be explained from the perspectives of informational advantages and supervisory effects. Interlocking directors, due to their positions on multiple company boards, can access and share information across industries, which helps enterprises identify and evaluate new investment opportunities, thus promoting diversified investments. Secondly, the interlocking director network may serve to mitigate the agency costs and risks associated with diversified investments. This is achieved by enhancing supervision and information flow, which in turn makes enterprises more willing to attempt diversification.

**Table 6:** decision centralization on internal capital market embedded in interlocking director network.

	(1)	(2)
VARIABLES	Icm__d_	Icm__e_
CI	-0.318^***^	0.077^*^
	(0.122)	(0.044)
Network_d	0.456^***^	0.063^**^
	(0.082)	(0.027)
c.CI × c.Network_d	0.155	0.773^**^
	(0.884)	(0.316)
size	0.063^***^	0.001
	(0.004)	(0.001)
roa	-0.536^***^	0.008
	(0.076)	(0.026)
top1	-0.031	-0.075^***^
	(0.025)	(0.009)
lev	0.205^***^	-0.004
	(0.024)	(0.009)
growth	0.016^***^	-0.002
	(0.004)	(0.002)
BoardScale	0.004^**^	0.003^***^
	(0.002)	(0.001)
tobin	0.012^***^	-0.006^***^
	(0.004)	(0.001)
EU	0.009^***^	0.006^***^
	(0.003)	(0.001)
Constant	-1.033^***^	0.664^***^
	(0.082)	(0.030)
Observations	16,997	16,997
R-squared	0.154	0.075
Year FE	Yes	Yes
Indu FE	Yes	Yes

Robust standard errors in parentheses

*** p < 0.01,

** p < 0.05,

* p < 0.1

***3.2.2.2 The moderating effect of interlocking director network on the impact of decision centralization and internal capital market efficiency***. Table 6, column (2), shows that after following the incorporation of the director network as a moderator, the interaction term c.CI × c.Network_d after centering the variables, exhibits a coefficient of 0.063, which is statistically significant significant at the 5% level. This indicates the influence of decision centralization (CI) on internal capital market efficiency (Icm_e). These findings indicate that the interlocking director network has, to some extent, augmented the positive impact of decision centralization on internal capital market efficiency. Consequently, Hypothesis 3.2 is not supported by the data. This suggests that the interlocking director network may play a role in improving the efficiency of the internal capital market through the sharing of information and the provision of supervision. The findings suggest that the concentration of decision-making authority among corporate managers contributes to the efficiency of the internal capital market. Furthermore, the impact of the interlocking director network is aligned with the objectives of the internal decision-making layer, namely, the goals of increased efficiency and optimized resource allocation within the group. This alignment may be attributed to the interlocking director network’s capacity to mitigate agency costs and risks through supervision and information dissemination, thereby enhancing the operational efficiency of the internal capital market. At the same time, column (1) shows that the coefficient for Network_d is 0.456, in comparison to the coefficient for Network_e, which is 0.063. This indicates that the interlocking director network itself exhibits a pronounced tendency to facilitate diversification. This reveals that the interlocking director network may serve a more substantial role as an information disseminator within the internal capital market, particularly with regard to diversified investments. By establishing connections between disparate entities, the interlocking director network can furnish cross-industry market and industry data, aiding enterprises in identifying and assessing novel investment prospects, thereby facilitating diversified investments.

#### 3.2.3 Analysis of the impact of internal capital market on risk-taking embedded in associated institutional investor network.

***3.2.3.1 The moderating effect of associated institutional investor on the impact of internal capital market diversification and risk-taking.*** Table 7, column (1), illustrates the moderating effect of the associated institutional investor network (Network_i) on the relationship between internal capital market diversification (Icm_d) and corporate risk-taking. In the model, the coefficient for the interaction term c.Icm_d × c.Network_i is 0.035, which is significant at the 1% level. This indicates that the associated institutional investor network strengthens the positive impact of internal capital market diversification on risk-taking. Similarly, a reduction in diversification levels is associated with a decline in enterprise risk-taking. The results of the test support Hypothesis4.1 is supported. Institutional investors, as informed traders, are unable to directly influence internal capital allocation decisions at the enterprise level through their own decision-making processes. However, they can indirectly affect risk-taking by arbitraging with private information, integrating company-specific information into stock prices and thereby influencing stock prices and risk-taking. Institutional investors contribute to a reduction in company risk-taking by hedging noise trading.

**Table 7:** Internal capital market on risk-taking embedded in associated institutional investor network.

	(1)	(2)
VARIABLES	risk	risk
Icm_d	0.034^***^	
	(0.004)	
Network_i	-0.008	
	(0.007)	
c.Icm_d × c.Network_i	0.035^***^	
	(0.008)	
Icm_e		-0.110^***^
		(0.013)
Network_i		0.093^***^
		(0.018)
c.Icm_e × c.Network_i		-0.126^***^
		(0.026)
size	-0.054^***^	-0.050^***^
	(0.002)	(0.002)
roa	-0.092^**^	-0.122^***^
	(0.037)	(0.037)
top1	-0.055^***^	-0.068^***^
	(0.011)	(0.012)
lev	0.206^***^	0.213^***^
	(0.011)	(0.011)
growth	0.005^**^	0.005^**^
	(0.002)	(0.002)
BoardScale	-0.004^***^	-0.004^***^
	(0.001)	(0.001)
tobin	0.034^***^	0.034^***^
	(0.002)	(0.002)
EU	0.004^***^	0.005^***^
	(0.001)	(0.001)
Constant	2.983^***^	2.982^***^
	(0.042)	(0.043)
Observations	16,997	16,997
R-squared	0.520	0.522
Year FE	Yes	Yes
Indu FE	Yes	Yes

Robust standard errors in parentheses

*** p < 0.01,

** p < 0.05,

* p < 0.1

***3.2.3.2 The moderating effect of associated institutional investor on the impact of internal capital market efficiency and risk-taking*.** Table 7, column (2), demonstrates the moderating effect of the associated institutional investor network (Network_i) on the relationship between internal capital market efficiency (Icm_e) and corporate risk-taking. In the model, the coefficient for the interaction term c.Icm_e × c.Network_i is -0.126, which is significant at the 1% level. This indicates that the associated institutional investor network strengthens the reducing impact of internal capital market efficiency on risk-taking. This result lends support to Hypothesis Hypothesis4.2, which suggests that the associated institutional investor network may convey positive signals about the enterprise through its reputation mechanism and signal transmission function. This, in turn, increases the efficiency of the internal capital market and suppresses stock price fluctuations, which in turn reduces the enterprises’ risk-taking. This may be attributed to the fact that the signals conveyed by associated institutional investors through their network can influence corporate behavior and market performance, thereby enhancing the efficiency of the internal capital market and reducing risk. Furthermore, it is evident that the absolute value of the coefficient influenced by the reputation mechanism and signal transmission mechanism of institutional investors is larger (0.126–0.035), indicating that, in comparison to the uncertainty introduced by diversification, institutional investors are more likely to achieve a favorable reputation and stabilize stock prices by enhancing the efficiency of the internal capital market.

### 3.3 Further analysis and inspection

#### 3.3.1 Based on risk nature.

In the main regression, we discuss the overall risk level of enterprises. Based on the nature of risk, we can further divide the risk into external risk and the enterprise’s own risk. We employ the Capital Asset Pricing Model (CAPM) [61]to decompose the overall risk into its systemic and idiosyncratic components. The model is specified as follows:

*R*_*i,t*_*=α+βR*_*m,t*_*+∊*_*i,t*_, where *R*_*m,t*_ represents the market’s comprehensive return rate on day t. Systemic risk is captured by the coefficient β, indicating the enterprise’s sensitivity to market movements. Idiosyncratic risk is represented by the residual term ∊_i,t_, which is the deviation of the enterprise’s risk from the market risk. To facilitate interpretation and enhance data readability, the systemic risk variable, designated “system_risk” is calculated as *ln(250×β*_*i*_*).* Similarly, the idiosyncratic risk variable “idiosyn_risk” is determined by calculating the standard deviation of the residual term ∊ and applying the same transformation, resulting in *ln(250 × std(*∊_*i,t*_*)).* Regression results presented in Table 8,columns (1) and (2), indicate that the coefficient for corporate centralization of decision-making (CI) on idiosyncratic risk (idiosyn_risk) is -0.072 and -0.231, respectively. both of which are significant at the 1% level. This suggests that corporate centralization of decision-making is significantly associated with a reduction in idiosyncratic risk. It may imply that a centralized decision-making structure may be more effective in managing risk factors specific to the enterprise. For systemic risk (system_risk), the coefficients for CI are -0.021 (column 3) and -0.035 (column 4). While these figures are not statistically significant, they do exhibit a discernible pattern of association with the variable representing idiosyncratic risk. This indicates that centralization may also have some impact on systemic risk, suggesting that the allocation of decision-making authority within enterprises may suppress their own risk-taking, but its role in mitigating systemic risk is constrained. Systemic risk is typically associated with macroeconomic factors and overall market conditions, which are beyond the control of individual enterprises. Consequently, when managing risk, enterprises must not only optimize their internal decision-making structures but also consider changes in the external market environment and adopt appropriate hedging strategies to manage systemic risk.

**Table 8:** The Impact of Corporate Decision-Making Centralization on Idiosyncratic and Systematic Risks.

	(1)	(2)	(3)	(4)
VARIABLES	idiosyn_risk	idiosyn_risk	system_risk	system_risk
CI	-0.072^***^	-0.231^***^	-0.021	-0.035
	(0.026)	(0.024)	(0.029)	(0.029)
size		-0.068^***^		-0.020^***^
		(0.002)		(0.003)
roa		-0.068		0.128^**^
		(0.044)		(0.064)
top1		-0.012		-0.139^***^
		(0.014)		(0.016)
lev		0.288^***^		0.090^***^
		(0.014)		(0.017)
growth		0.009^***^		-0.000
		(0.002)		(0.003)
BoardScale		-0.005^***^		-0.002
		(0.001)		(0.002)
tobin		0.051^***^		0.000
		(0.002)		(0.003)
EU		0.009^***^		-0.012^***^
		(0.002)		(0.002)
Constant	1.693^***^	3.029^***^	5.570^***^	6.058^***^
	(0.002)	(0.050)	(0.002)	(0.068)
Observations	16,997	16,997	16,988	16,988
R-squared	0.318	0.426	0.309	0.317
Year FE	Yes	Yes	Yes	Yes
Indu FE	Yes	Yes	Yes	Yes

Robust standard errors in parentheses

*** p < 0.01,

** p < 0.05,

* p < 0.1

#### 3.3.2 Based on enterprise nature.

In China, state-owned enterprises (SOEs) constitute a fundamental pillar of the national economy. Concurrently, private enterprises constitute a significant component of the economic landscape, exemplifying competitive spirit and entrepreneurial dynamism. Different ownership structures have different decision-making processes in authority allocation and risk management. The impact of centralized decision-making (CI) on risk-taking (risk) in state-owned and private enterprises is illustrated in Columns (1) and (2) of Table 9, respectively. The regression results indicate that the coefficient for centralized decision-making (CI) for state-owned enterprises is -0.179, while for private enterprises, it is -0.154. Both coefficients are statistically significant at the 1% level, suggesting that the concentration of decision-making authority is significantly associated with a reduction in risk-taking for both state-owned and private enterprises. Furthermore, state-owned enterprises demonstrate a more pronounced downward trend in the impact of centralized decision-making (CI) on risk-taking (risk). This may be attributed to the fact that state-owned enterprises are typically under the direct or indirect control of the government, and government authorization may result in a more centralized decision-making process. The centralized decision-making structure may encourage state-owned enterprises to exercise greater caution when confronted with risks, as decisions are not solely driven by corporate interests but also encompass the safeguarding and enhancement of state-owned assets and national economic security. In contrast, private enterprises, due to the concentration of ownership and control, often possess more flexible and efficient decision-making processes and may be more inclined to assume risks in pursuit of higher returns.

**Table 9:** Analysis of the Heterogeneity of Centralized Management and Risk-Taking.

	(1)	(2)	(3)	(4)	(5)	(6)
	state-owned	private	Profit orientation	Non-profit orientation	Growth orientation	Non-growth orientation
VARIABLES	risk	risk	risk	risk	risk	risk
CI	-0.179^***^	-0.154^***^	-0.102^***^	-0.157^***^	-0.145^***^	-0.149^***^
	(0.033)	(0.026)	(0.039)	(0.024)	(0.055)	(0.022)
size	-0.060^***^	-0.034^***^	-0.057^***^	-0.049^***^	-0.031^***^	-0.054^***^
	(0.003)	(0.003)	(0.004)	(0.002)	(0.005)	(0.002)
roa	0.071	-0.230^***^	-0.060	-0.050	-0.089	-0.044
	(0.063)	(0.045)	(0.085)	(0.041)	(0.093)	(0.040)
top1	-0.023	-0.017	-0.014	-0.025^*^	-0.019	-0.038^***^
	(0.018)	(0.015)	(0.022)	(0.014)	(0.031)	(0.012)
lev	0.312^***^	0.141^***^	0.211^***^	0.177^***^	0.100^***^	0.218^***^
	(0.018)	(0.015)	(0.024)	(0.013)	(0.029)	(0.012)
growth	0.004	0.006^**^	-0.003	0.006^***^	0.013^***^	0.003
	(0.003)	(0.003)	(0.005)	(0.002)	(0.004)	(0.002)
BoardScale	-0.003^*^	-0.003^*^	-0.003	-0.002^**^	-0.004	-0.003^**^
	(0.001)	(0.002)	(0.002)	(0.001)	(0.003)	(0.001)
tobin	0.043^***^	0.034^***^	0.041^***^	0.033^***^	0.042^***^	0.035^***^
	(0.003)	(0.002)	(0.003)	(0.002)	(0.005)	(0.002)
EU	0.006^**^	0.004^**^	0.003	0.002	0.001	0.004^***^
	(0.002)	(0.002)	(0.003)	(0.002)	(0.003)	(0.001)
Constant	3.025^***^	2.582^***^	2.993^***^	2.892^***^	2.554^***^	2.959^***^
	(0.059)	(0.062)	(0.094)	(0.046)	(0.108)	(0.044)
Observations	7,251	9,745	4,608	12,387	2,614	14,381
R-squared	0.549	0.491	0.588	0.492	0.488	0.528
Year FE	Yes	Yes	Yes	Yes	Yes	Yes
Indu FE	Yes	Yes	Yes	Yes	Yes	Yes

Robust standard errors in parentheses

*** p < 0.01,

** p < 0.05,

* p < 0.1

#### 3.3.3 Based on decision preferences.

The efficacy of a centralized management model is significantly shaped by the preferences of managers, As the foundation for enterprises to formulate strategic decisions, strategic orientation fundamentally affects business activities and strategies, and thus also influences the motivation and propensity of enterprises to assume risk taking, particularly when their decisions diverge from the interests of shareholders or other stakeholders. This study references the work of Liu Xia (2023) [62], which employs the net profit margin of total assets and the growth rate of total assets as indicators of a company’s profitability and growth capacity, respectively. The methodology entails calculating the average profitability and growth capacity of enterprises over the sample period and comparing these metrics to industry averages. An enterprise is classified as profit-oriented if its profitability exceeds the industry average, which is assigned a value of 1; otherwise, it is given a value of 0. Conversely, if an enterprise’s growth capacity is below the industry norm, it is deemed growth-oriented, and is assigned a value of 1; otherwise, it receives a 0. The analyze is presented in Columns (3) to (6) of Table 9. The coefficients of CI are all negative, indicating that regardless of the strategic orientation of the enterprise, centralized decision-making is associated with lower risk-taking. From columns (3) and (4), which focus on short-term profits, the CI coefficient for column (3) (Profit-oriented) is -0.102, and for column (4) (non-profit-oriented) is -0.157. The greater the absolute value of the CI coefficient for non-profit-oriented enterprises, it can be inferred that the impact of centralized decision-making on risk reduction is more pronounced in such enterprises. This may be related to the fact that non-profit-oriented enterprises tend to prioritize long-term objectives and social impact, leading to a greater inclination towards risk aversion and more cautious decision-making. From columns (5) and (6), which focus on long-term growth, the CI coefficient for column (5) (Growth-oriented) is -0.145, and for column (6) (non-growth-oriented) is -0.149. The CI coefficient for growth-oriented enterprises is slightly lower in absolute value than that for non-growth-oriented enterprises, which may imply that non-growth-oriented enterprises may pay more attention to risk control in centralized decision-making. The above analysis indicates that profit-oriented and growth-oriented enterprises may focus more on financial performance and market expansion, while non-profit-oriented and non-growth-oriented enterprises may pay more attention to stable operations and social impact.

## 4 .Conclusion

### 4.1. Conclusions and recommendations

The findings of this study highlight the critical function of decision-making authority within the internal capital market and its mediation through the correlation network. In light of the aforementioned analysis, the paper presents a number of insights that may prove beneficial in both practical applications and future research endeavors.

Firstly, it is imperative to establish a balanced mechanism that strikes a balance between “centralization” and “decentralization” to optimize resource allocation. It would be prudent for enterprise groups to enhance their risk-taking capacity in order to mitigate potential risk contagion. The concentration of authority within enterprise groups facilitates more optimal resource allocation and mitigates the overall risk exposure of the group. However, this approach may also result in the forfeiture of certain investment opportunities. The operation of enterprise centralization has the effect of suppressing the flexibility of the group and the initiative of subsidiaries. However, it also serves to avoid the contagion of risks.

Secondly, it is of paramount importance to optimize the shareholding structure. Empirical evidence indicates that private capital frequently centralizes management for the purpose of risk control. The involvement of state-owned capital may serve to mitigate the risk of stock price collapses in private enterprises. The mixed-ownership reform has the potential to enhance risk aversion and alleviate information asymmetry, particularly by curbing the opportunistic behavior of controlling shareholders. This is particularly relevant in the context of private enterprises, where governance mechanisms are often inadequate and management demonstrates a low propensity for risk aversion and is subject to limited external oversight.

Thirdly, the promotion of a stakeholder joint governance mechanism is recommended. By integrating the system, ownership, organizational, and internal control levels, a cohesive governance framework can be established. This approach ensures that the interests of all stakeholders are aligned, thereby maximizing enterprise value.

In conclusion, the strategic distribution of decision-making authority within enterprise groups, when viewed in the context of their internal capital markets and associated network, is of paramount importance for effective risk management and value creation. The insights presented in this paper contribute to a more comprehensive understanding of corporate governance and risk-taking behaviors within enterprise groups. For all tables and figures, please refer to the Supporting Information S1 Table, S2 Fig

### 4.2. Limitations of this study

The present study is subject to a number of limitations that require further investigation and resolution in future research. The data sample of this study is limited to listed companies in China, which may limit the generalizability of the research findings in other global environments or private companies. The decision-making authority allocation and risk-taking behavior of enterprise groups may be influenced by factors such as market structures, regulatory environments, and cultural differences across countries.

Moreover, the dataset excluded financial companies, which may limit the broad applicability of the research conclusions. It is possible that companies in the financial sector may exhibit different features in internal capital markets and risk-taking due to their unique industry characteristics and risk management practices.

Additionally, although this study analyzed the internal capital market using two different dimensions—efficiency and diversification—the data relied on public financial data of listed companies, which may not fully capture the entire dynamics and complexity of the internal capital market. It would be beneficial for future research to consider using more detailed internal transaction data to gain deeper insights.

## Supporting information

S1 Table This table in the paper includes empirical tables. (PDF)

S2 Fig This figure represents the diagram of variable relationships. (PDF)

S3 Data It Contains all 16,997 observations of this study, including the variables involved in the paper. (XLSX)
