# Supplementary material for: The associated network embedded decision-making authority allocation and risk-taking of enterprise groups
Source: PLoS One. 2025 May 8;20(5):e0318983. doi: 10.1371/journal.pone.0318983 (PMC12061140; doi:10.1371/journal.pone.0318983)
Supplement: S1 Table — (PDF) [file pone.0318983.s001.pdf]

Table 1. Definitions and Descriptions of Main Variables

variable definition	*variable symbol*		*variable declaration*
Interpreted variable	*risk*		*Total risk: the standard deviation of the annual daily return rate of the listed company*
Interpretive variable	*CI*		*Degree of capital centralization within the enterprise group:* $PSalary_{i,t}=\beta 0+\beta 1PAsset_{i,t}+\varepsilon$ *The model shown performs annual regression and takes the estimated residual as a measure of the degree of centralization*
mediating variable	*Icm*	Icm_d	*Operation mode of internal capital market: revenue entropy index* $Icm_d = P_i ln\ (1/ P_i)$, P_i *= main business income/total business income of enterprise category I. The larger the* Icm_d*, the more diversified the internal capital market is.*
		Icm_e	*Efficiency of internal capital market within the enterprise group* $Icm_{ei,t}=\alpha_0+\alpha 1Icm_{ei,t-1}+\alpha_2TobinQ_{i,t-1}+\alpha_3Cash_{i,t-1}+\alpha_4Lev_{i,t-1}+\alpha_5Size_{i,t-1}+\alpha_6Roa_{i,t-1}+\alpha_7IPO_{i,t-1}+\lambda_i+\mu_t+\epsilon_{i,t}$ *and the larger the absolute value, the higher the efficiency.*
Adjustment variables	*Network*	$Network_d$	*The proximity of the director network reflects the proximity between a director and other directors in the network. For a director, the closer he is to other directors, the greater the proximity center, and the more obvious the information advantage between the directors*
		$Network_i$	*The proximity of the institutional investor network reflects the proximity between an institutional investor and other institutional investors in the network. For an institutional investor, the closer it is to other institutional investors, the greater the proximity center, and the more pronounced the information advantage between the institutional investors.*
Control variables	*Size*		*Total assets taken to the natural logarithm.*
	TobinQ		*Total market value / assets*
	Growth		*Current operating income / previous operating income of-1*
	lev		*Interest-bearing liabilities / total assets*
	roa		*EBITDA / total assets*
	top1		*The largest shareholder shareholding ratio*
	BoardScale		*The board of directors' size (number of members)*
	EU		*The standard deviation of the residuals of operating income adjusted for the industry over the past 5 years*
	Year		Annual virtual variable
	Industry		Industry virtual variable

Table 2: Descriptive Statistics Table

Variable	Obs	Mean	Std. Dev.	Min	Max
risk	16997	1.892	0.29	1.146	2.589
CI	16997	-0.01	0.082	-0.216	0.171
Icm d	16997	0.6	0.474	0	1.466
Icm e	16997	0.683	0.156	0.458	0.997

Network d	16997	0.133	0.043	0	0.186
Network i	16997	0.211	0.359	0	1
size	16997	22.612	1.329	20.084	26.413
roa	16997	0.039	0.055	-0.183	0.198
top1	16997	0.355	0.15	0.096	0.75
lev	16997	0.461	0.196	0.074	0.885
growth	16997	0.331	0.921	-0.662	6.521
BoardScale	16997	8.717	1.693	5	15
tobin	16997	1.96	1.265	0.834	8.224
EU	16997	1.252	1.383	0.083	8.446

Table 3: Test Results of the Influence of Enterprise Group Decision-making authority on Risk-Taking

	(1)	(2)	(3)	(4)		(5)	(6)
VARIABLES	risk	risk	risk	risk	VARIABLES	risk	risk
CI	-0.065***	-0.175***	-0.067***	-0.179***	L.CI	-0.024	-0.147***
	(0.021)	(0.020)	(0.021)	(0.020)		(0.024)	(0.022)
size		-0.052***		-0.052***	size		-0.050***
		(0.002)		(0.002)			(0.002)
roa		-0.095***		-0.095***	roa		-0.131***
		(0.037)		(0.034)			(0.041)
top1		-0.045***		-0.045***	top1		-0.056***
		(0.012)		(0.012)			(0.013)
lev		0.221***		0.221***	lev		0.245***
		(0.011)		(0.011)			(0.013)
growth		0.005***		0.005***	growth		0.006***
		(0.002)		(0.002)			(0.002)
BoardScale		-0.004***		-0.004***	BoardScale		-0.004***
		(0.001)		(0.001)			(0.001)
tobin		0.035***		0.035***	tobin		0.040***
		(0.002)		(0.002)			(0.002)
EU		0.004***		0.004***	EU		0.006***
		(0.001)		(0.001)			(0.002)
Constant	1.891***	2.942***	2.016***	2.965***	Constant	1.882***	2.880***
	(0.002)	(0.042)	(0.029)	(0.047)		(0.002)	(0.046)
Observations	16,997	16,997	16,997	16,997	Observations	14,482	14,482
R-squared	0.446	0.517	0.446	0.517	R-squared	0.452	0.524
Year FE	Yes	Yes	Yes	Yes	Year FE	Yes	Yes
Indu FE	Yes	Yes	Yes	Yes	Indu FE	Yes	Yes

Robust standard errors in parentheses

*** p<0.01, ** p<0.05, * p<0.1

Table 4: Regression Results of the Internal Capital Market and Enterprise Financial Crisis Model

	(1)	(2)	(3)	(4)
VARIABLES	zscore	zscore	risk	risk
CI	-0.338***	-0.694***		
	(0.106)	(0.110)		
GROUP			-0.044***	-0.020***
			(0.004)	(0.004)
size		-0.136***		-0.047***
		(0.009)		(0.002)
roa		-0.585***		-0.134***
		(0.185)		(0.037)
top1		0.152**		-0.046***
		(0.060)		(0.011)
lev		0.035		0.210***
		(0.090)		(0.011)
growth		-0.034***		0.005***
		(0.010)		(0.002)
BoardScale		-0.001		-0.003***
		(0.006)		(0.001)
tobin		0.144***		0.035***
		(0.017)		(0.002)
EU		0.005		0.004***
		(0.009)		(0.001)
Constant	4.740***	7.487***	1.912***	2.839***
	(0.007)	(0.185)	(0.002)	(0.041)
Observations	16,997	16,997	16,997	16,997
R-squared	0.171	0.227	0.448	0.513
Year FE	Yes	Yes	Yes	Yes
Indu FE	Yes	Yes	Yes	Yes

Robust standard errors in parentheses

*** p<0.01, ** p<0.05, * p<0.1

Table5 The mediating role of the internal capital market in the relationship between decision-making authority allocation and risk-taking

	(2)	(3)	(4)	(5)
VARIABLES	icm$_{_d}$	risk	icm$_{_e}$	risk
CI	-0.307***	-0.162***	0.178***	-0.150***
	(0.043)	(0.020)	(0.015)	(0.020)
Icm_d		0.043***		
		(0.003)		

Icm_e				-0.144***
				(0.010)
size	0.065***	-0.055***	0.001	-0.052***
	(0.004)	(0.002)	(0.001)	(0.002)
roa	-0.538***	-0.072*	0.007	-0.094**
	(0.076)	(0.037)	(0.026)	(0.037)
top1	-0.030	-0.044***	-0.075***	-0.056***
	(0.025)	(0.011)	(0.009)	(0.012)
lev	0.206***	0.212***	-0.004	0.221***
	(0.024)	(0.011)	(0.009)	(0.011)
growth	0.016***	0.005**	-0.002	0.005**
	(0.004)	(0.002)	(0.002)	(0.002)
BoardScale	0.005**	-0.004***	0.003***	-0.003***
	(0.002)	(0.001)	(0.001)	(0.001)
tobin	0.013***	0.035***	-0.006***	0.035***
	(0.004)	(0.002)	(0.001)	(0.002)
EU	0.008***	0.004***	0.006***	0.005***
	(0.003)	(0.001)	(0.001)	(0.001)
Constant	-1.026***	2.987***	0.665***	3.038***
	(0.082)	(0.042)	(0.030)	(0.042)
Observations	16,997	16,997	16,997	16,997
R-squared	0.153	0.522	0.074	0.523
Year FE	Yes	Yes	Yes	Yes
Indu FE	Yes	Yes	Yes	Yes

Robust standard errors in parentheses

*** p<0.01, ** p<0.05, * p<0.1

Table6 decision centralization on internal capital market embedded in interlocking director network

	(1)	(2)
VARIABLES	Icm_{d}	Icm_{e}
CI	-0.318***	0.077*
	(0.122)	(0.044)
Network_d	0.456***	0.063**
	(0.082)	(0.027)
c.CI×c.Network_d	0.155	0.773**
	(0.884)	(0.316)
size	0.063***	0.001
	(0.004)	(0.001)
roa	-0.536***	0.008
	(0.076)	(0.026)
top1	-0.031	-0.075***

	(0.025)	(0.009)
lev	0.205***	-0.004
	(0.024)	(0.009)
growth	0.016***	-0.002
	(0.004)	(0.002)
BoardScale	0.004**	0.003***
	(0.002)	(0.001)
tobin	0.012***	-0.006***
	(0.004)	(0.001)
EU	0.009***	0.006***
	(0.003)	(0.001)
Constant	-1.033***	0.664***
	(0.082)	(0.030)
Observations	16,997	16,997
R-squared	0.154	0.075
Year FE	Yes	Yes
Indu FE	Yes	Yes

Robust standard errors in parentheses

*** p<0.01, ** p<0.05, * p<0.1

Table7 Internal capital market on risk-taking embedded in associated institutional investor network

	(1)	(2)
VARIABLES	risk	risk
Icm_d	0.034***	
	(0.004)	
Network_i	-0.008	
	(0.007)	
c.Icm_d×c.Network_i	0.035***	
	(0.008)	
Icm_e		-0.110***
		(0.013)
Network_i		0.093***
		(0.018)
c.Icm_e×c.Network_i		-0.126***
		(0.026)
size	-0.054***	-0.050***
	(0.002)	(0.002)
roa	-0.092**	-0.122***
	(0.037)	(0.037)
top1	-0.055***	-0.068***

	(0.011)	(0.012)
lev	0.206***	0.213***
	(0.011)	(0.011)
growth	0.005**	0.005**
	(0.002)	(0.002)
BoardScale	-0.004***	-0.004***
	(0.001)	(0.001)
tobin	0.034***	0.034***
	(0.002)	(0.002)
EU	0.004***	0.005***
	(0.001)	(0.001)
Constant	2.983***	2.982***
	(0.042)	(0.043)
Observations	16,997	16,997
R-squared	0.520	0.522
Year FE	Yes	Yes
Indu FE	Yes	Yes

Robust standard errors in parentheses

*** p<0.01, ** p<0.05, * p<0.1

Table8 The Impact of Corporate Decision-Making Centralization on Idiosyncratic and Systematic Risks

	(1)	(2)	(3)	(4)
VARIABLES	idiosyn_risk	idiosyn_risk	system_risk	system_risk
CI	-0.072***	-0.231***	-0.021	-0.035
	(0.026)	(0.024)	(0.029)	(0.029)
size		-0.068***		-0.020***
		(0.002)		(0.003)
roa		-0.068		0.128**
		(0.044)		(0.064)
top1		-0.012		-0.139***
		(0.014)		(0.016)
lev		0.288***		0.090***
		(0.014)		(0.017)
growth		0.009***		-0.000
		(0.002)		(0.003)
BoardScale		-0.005***		-0.002
		(0.001)		(0.002)
tobin		0.051***		0.000

		(0.002)		(0.003)
EU		0.009***		-0.012***
		(0.002)		(0.002)
Constant	1.693***	3.029***	5.570***	6.058***
	(0.002)	(0.050)	(0.002)	(0.068)
Observations	16,997	16,997	16,988	16,988
R-squared	0.318	0.426	0.309	0.317
Year FE	Yes	Yes	Yes	Yes
Indu FE	Yes	Yes	Yes	Yes

Robust standard errors in parentheses

*** p<0.01, ** p<0.05, * p<0.1

Table 9: Analysis of the Heterogeneity of Centralized Management and Risk-Taking

	(1)	(2)	(3)	(4)	(5)	(6)
	state-owned	private	Profit orientation	Non-profit orientation	Growth orientation	Non-growth orientation
VARIABLES	risk	risk	risk	risk	risk	risk
CI	-0.179***	-0.154***	-0.102***	-0.157***	-0.145***	-0.149***
	(0.033)	(0.026)	(0.039)	(0.024)	(0.055)	(0.022)
size	-0.060***	-0.034***	-0.057***	-0.049***	-0.031***	-0.054***
	(0.003)	(0.003)	(0.004)	(0.002)	(0.005)	(0.002)
roa	0.071	-0.230***	-0.060	-0.050	-0.089	-0.044
	(0.063)	(0.045)	(0.085)	(0.041)	(0.093)	(0.040)
top1	-0.023	-0.017	-0.014	-0.025*	-0.019	-0.038***
	(0.018)	(0.015)	(0.022)	(0.014)	(0.031)	(0.012)
lev	0.312***	0.141***	0.211***	0.177***	0.100***	0.218***
	(0.018)	(0.015)	(0.024)	(0.013)	(0.029)	(0.012)
growth	0.004	0.006**	-0.003	0.006***	0.013***	0.003
	(0.003)	(0.003)	(0.005)	(0.002)	(0.004)	(0.002)
BoardScale	-0.003*	-0.003*	-0.003	-0.002**	-0.004	-0.003**
	(0.001)	(0.002)	(0.002)	(0.001)	(0.003)	(0.001)
tobin	0.043***	0.034***	0.041***	0.033***	0.042***	0.035***
	(0.003)	(0.002)	(0.003)	(0.002)	(0.005)	(0.002)
EU	0.006**	0.004**	0.003	0.002	0.001	0.004***
	(0.002)	(0.002)	(0.003)	(0.002)	(0.003)	(0.001)
Constant	3.025***	2.582***	2.993***	2.892***	2.554***	2.959***
	(0.059)	(0.062)	(0.094)	(0.046)	(0.108)	(0.044)
Observations	7,251	9,745	4,608	12,387	2,614	14,381
R-squared	0.549	0.491	0.588	0.492	0.488	0.528
Year FE	Yes	Yes	Yes	Yes	Yes	Yes
Indu FE	Yes	Yes	Yes	Yes	Yes	Yes

Robust standard errors in parentheses

*** p<0.01, ** p<0.05, * p<0.1
