# Supplementary material for: The associated network embedded decision-making authority allocation and risk-taking of enterprise groups
Source: PLoS One. 2025 May 8;20(5):e0318983. doi: 10.1371/journal.pone.0318983 (PMC12061140; doi:10.1371/journal.pone.0318983)
Supplement: S2 Fig — (PDF) [file pone.0318983.s002.pdf]

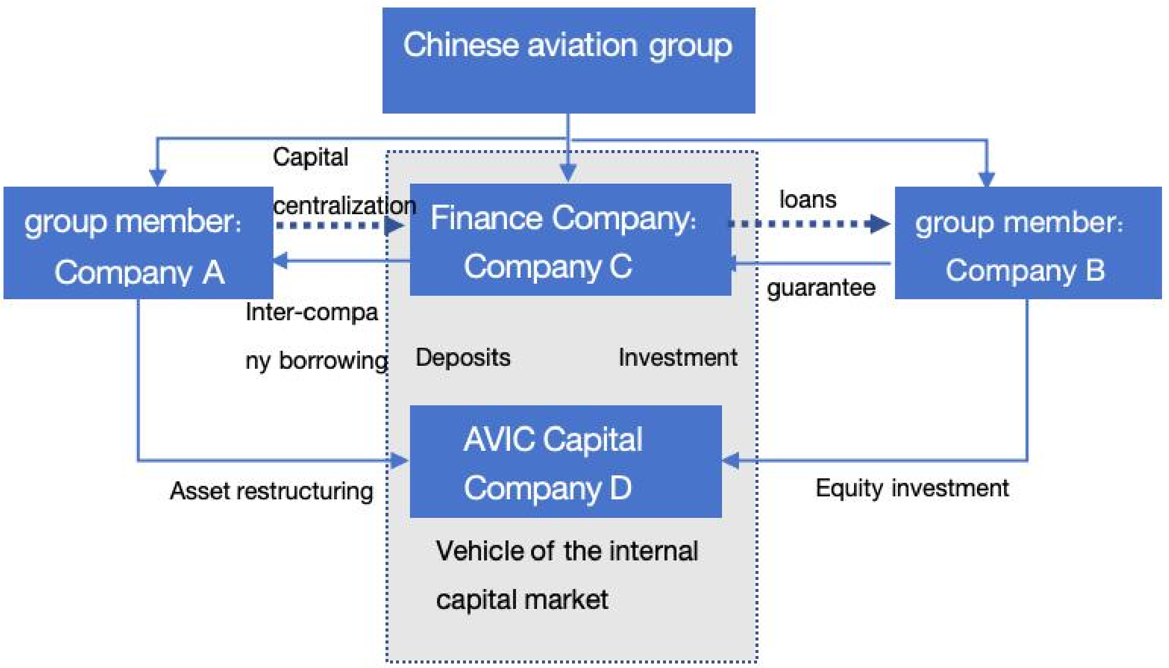

Figure1 The internal capital market decision-making and operation of China National Aviation Group

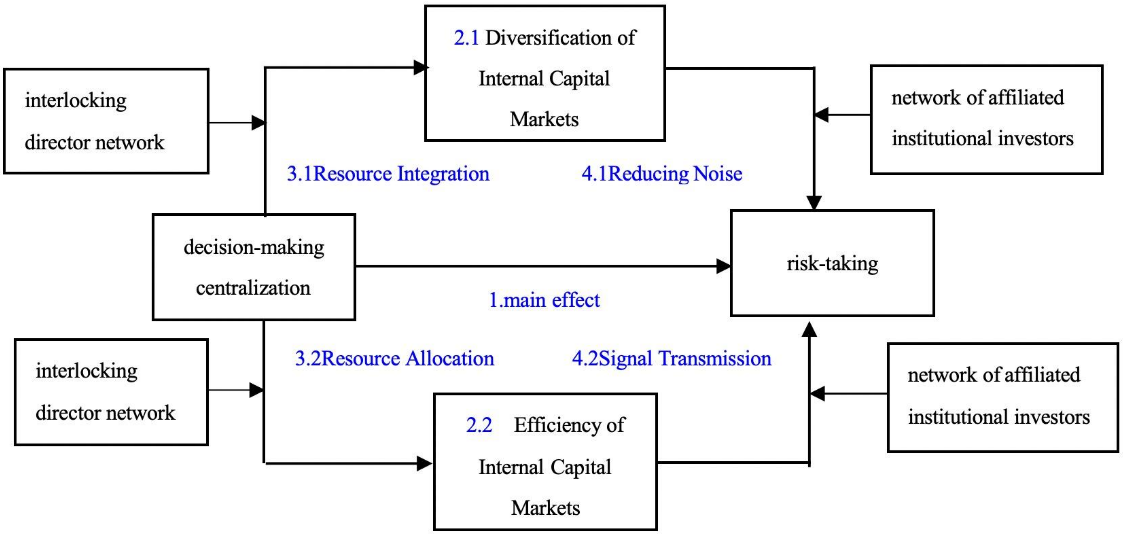

Figure2 Corporate group decision-making and risk effects based on internal affiliation network

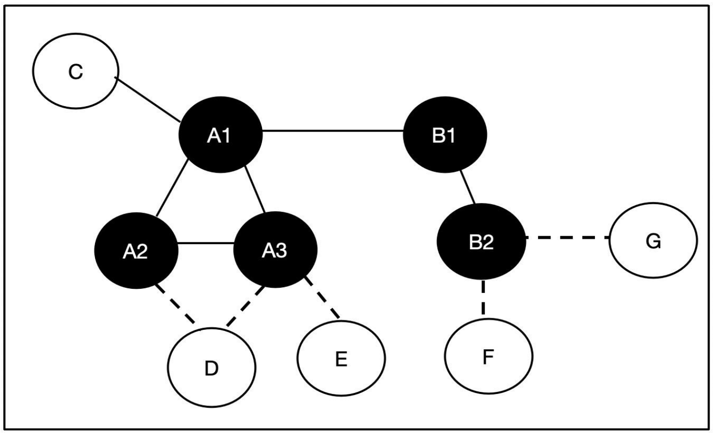

Figure3 Calculation of Key Indicators in the Associated Network
